# Adaptive Label Refinement Network for Domain Generalization in Compound Fault Diagnosis

**DOI:** 10.3390/s25226939

**Published:** 2025-11-13

**Authors:** Qiyan Du, Jiajia Yao, Jingyuan Yang, Fengmiao Tu, Suixian Yang

**Affiliations:** 1 School of Mechanical Engineering, Sichuan University, Chengdu 610065, China; duqiyan2001@163.com (Q.D.); yaojiajia@stu.scu.edu.cn (J.Y.); 2023323025024@stu.scu.edu.cn (F.T.); 2 School of Electrical and Electronic Engineering, Newcastle University, Newcastle upon Tyne NE1 7RU, UK

**Keywords:** compound fault diagnosis, domain generalization, adaptive label refinement, label refinement stability coefficient, convolutional neural network

## Abstract

**Highlights:**

**What are the main findings?**

- A novel label refinement framework is proposed for compound fault diagnosis under source-scarce conditions, which iteratively evolves soft labels to enhance domain generalization.
- A KL-divergence-based stability coefficient autonomously guides the iterative label refinement process.

**What is the implication of the main finding?**

- ALRN achieves over 22% accuracy gain, setting a new state-of-the-art under source-scarce conditions
- It offers a practical solution for industrial diagnosis where collecting diverse labeled data is prohibitive.

**Abstract:**

Domain generalization (DG) aims to develop models that perform robustly on unseen target domains, a critical but challenging objective for real-world fault diagnosis. The challenge is further complicated in compound fault diagnosis, where the rigidity of hard labels and the simplicity of label smoothing under-represent inter-class relations and compositional structures, degrading cross-domain robustness. While current domain generalization methods can alleviate these issues, they typically rely on multi-source domain data. However, considering the limitations of equipment operational conditions and data acquisition costs in industrial applications, only one or two independently distributed source datasets are typically available. In this work, an adaptive label refinement network (ALRN) was designed for learning with imperfect labels under source-scarce conditions. Compared to hard labels and label smoothing, ALRN learns richer, more robust soft labels that encode the semantic similarities between fault classes. The model first trains a convolutional neural network (CNN) to obtain initial class probabilities. It then iteratively refines the training labels by computing a weighted average of predictions within each class, using the sample-wise cross-entropy loss as an adaptive weighting factor. Furthermore, a label refinement stability coefficient based on the max-min Kullback–Leibler (KL) divergence ratio across classes is proposed to evaluate label quality and determine when to terminate the refinement iterations. With only one or two source domains for training, ALRN achieves accuracy gains exceeding 22% under unseen operating conditions compared with a conventional CNN baseline. These results validate that the proposed label refinement algorithm can effectively enhance the cross-domain diagnostic performance, providing a novel and practical solution for learning with imperfect supervision in cross-domain compound fault diagnosis.

## 1. Introduction

Condition monitoring and fault diagnosis of mechanical equipment are crucial for ensuring the safety and reliability of modern industrial systems [1,2,3]. In this field, vibration analysis utilizing accelerometer signals has been established as the most prevalent and mature methodology. The high signal-to-noise ratio of vibration signals and their direct relationship to mechanical dynamics make them particularly suitable for fault diagnosis [4,5,6]. However, the requirement for physical contact and precise sensor mounting can be a limitation in many scenarios. In comparison, the non-contact nature of acoustic analysis has garnered significant attention as a complementary technique, presenting unique advantages in scenarios where sensor installation is impractical or where faults generate distinct acoustic signatures [7,8,9]. Nevertheless, the accurate and robust fault identification using acoustic signals remains challenging because of their inherent non-stationarity and high susceptibility to environmental noise [10,11,12].

To address these challenges, deep learning models have been widely adopted to automatically learn discriminative features from complex signals. Various advanced architectures have been explored in the literature. Notably, graph neural networks (GNNs) have shown great promise in modeling the structural dependencies within mechanical systems for fault diagnosis, as evidenced by recent works on self-supervised graph feature enhancement (EG-SAGCN) [13], multi-sensor graph attention (MMHGAT) [14], and multiscale channel attention with graph fusion [15]. However, these methods typically require data from multiple sensors to construct meaningful graphs and capture the topological interactions among them.

In single-sensor scenarios, convolutional neural network (CNN) [16,17,18,19], by leveraging its local connectivity and weight-sharing mechanisms, combined with multi-layer convolution and pooling operations, can effectively capture local time-frequency features and global contextual information within signals. This has led to their increased application in fault diagnosis. Recently, attention mechanisms have also been integrated into CNN, guiding the models to prioritize salient fault-related information and thereby enhancing feature discrimination capabilities [20,21]. The core strength of CNN lies in its ability to hierarchically learn from structured data, effectively capturing essential patterns for accurate diagnosis. However, when distributional discrepancies exist between source and target domains due to variations in operating conditions, the non-stationary nature of acoustic signals exacerbates this issue, causing severe feature distribution mismatches [22,23]. This degrades the model generalization performance on unseen target domains and hinders the learning of cross-domain invariant features.

Domain adaptation (DA) has emerged as a critical methodology for resolving domain shift issues. Its fundamental objective is to learn invariant feature representations across source and target domains, thereby eliminating the adverse effects induced by distribution discrepancies [24,25,26]. Numerous methods have been developed to achieve this cross-domain alignment, including the Gaussian mixture variational-based transformer (GMVTDA) [27] and domain adversarial training (DAT) [28]. Furthermore, building upon maximum mean discrepancy (MMD), a novel discrepancy metric termed maximum mean square discrepancy (MMSD) was introduced to comprehensively capture both mean and variance discrepancies in the reproducing Kernel Hilbert space [29,30]. Despite these advancements, an inherent limitation of DA methods is their requirement for target domain data during training, regardless of label availability [31]. This poses a significant challenge for industrial applications, where obtaining target domain data is often difficult or expensive.

In contrast, domain generalization (DG) aims to generalize models trained on multiple source datasets to unseen target domains [32,33,34]. It achieves this by learning domain-invariant representations from the source domains alone, thereby eliminating the need for target data and offering broader applicability [35,36,37]. For example, domain-invariant features can be extracted through a multi-domain fusion generation module that incorporates cross-domain multivariate linearization [38]. DG also allows distributions to be more comprehensive by matching both joint distributions and domain-relevant distributions, rather than only marginal statistics [39]. Furthermore, data augmentation techniques like CycleGAN have been leveraged for domain generalization by synthesizing novel source domains to learn domain-invariant features [40,41,42].

While DG methods excel in mechanical fault diagnosis, their performance in compound fault scenarios is hindered by the inherent limitations of hard labels, often requiring multiple source domains for accurate detection. Considering the industrial scenario, only one or two source domains are typically available, which severely affects the applicability of existing methods. To overcome this challenge, techniques like label smoothing [43,44] have been employed. However, label smoothing’s assumption of uniform label distribution often leads to suboptimal performance in complex industrial environments [45,46]. To directly learn richer, more robust supervisory signals from scarce source domains, this paper proposes an adaptive label refinement network (ALRN). The primary objective is to develop a framework that autonomously evolves and enhances label supervision. This approach moves beyond static one-hot encodings to capture the rich, inter-class semantic relationships essential for generalizing to unseen domains. The core idea is implemented through an iterative process where a new soft label for each class is generated by a weighted aggregation of the model output probability distributions across all samples within that class. The weight assigned to each sample is determined by its training loss, ensuring that samples which are more challenging for the current model contribute more significantly to the refined label definition. This process is autonomously guided by a KL-divergence-based stability coefficient, which is employed as the convergence criterion to ensure refinement quality. The main contributions of this study are as follows:

1. This study reveals imperfect label supervision as a critical factor undermining cross-domain generalization performance under the challenging yet practical conditions of scarce source domains and prevalent compound faults.
2. A novel adaptive label refinement algorithm is proposed, through which soft labels are dynamically calibrated by an intra-class weighting mechanism. This process is autonomously guided by a KL-divergence-based stability coefficient, which is utilized to quantitatively monitor the refinement process and determine its convergence, thereby eliminating the need for a pre-defined iteration count.
3. Extensive experiments on a planetary gearbox compound fault dataset demonstrate that the proposed ALRN framework establishes a new state-of-the-art for cross-domain fault diagnosis, achieving a significant accuracy improvement against conventional supervised baselines in both single-source and dual-source settings.

## 2. Preliminaries

### 2.1. Domain Generalization Problem Statement

DG learns robust models from known source domains that can be directly transferred to operate effectively on unseen target domains. The learning process in the source domain can be described as finding a mapping function $f_{s}$ that operates on data pairs drawn from the source distribution:

(1) $$f_{s}:x^{s}\to y^{s},(x^{s},y^{s})∼P^{s}(X,Y)$$

where $x^{s}$ and $y^{s}$ denote a set of observed samples and their corresponding class labels drawn from the source domain input space $X^{s}$ and output space $Y^{s}$, and $P^{s}(X,Y)$ is the joint probability distribution of the source domain.

The goal of domain generalization is to leverage f to make accurate predictions on an unseen target domain:

(2) $$y^{t}=f_{s}(x^{t}),(x^{t},y^{t})∼P^{t}(X,Y),P^{t}(X,Y)\neq P^{s}(X,Y)$$

where $x^{t}$ denotes an input sample from the target domain, $y^{t}$ is its predicted label, and $P^{t}(X,Y)$ is the joint probability distribution of the target domain. This entails learning invariant patterns from limited source observations $\left\{(x^{s},y^{s})\right\}$ that preserve discriminative power under distributional shifts.

The establishment of transferable mappings from source to target domains is primarily governed by three critical factors: (1) the observational data $x^{s}$ in the source domain space, (2) the methodology for constructing the mapping function $f_{s}$, and (3) the quality of source-domain labels $y^{s}$. The precision of the data $x^{s}$ is inherently constrained by the data acquisition equipment. Prevailing DG methods aim to enhance cross-domain fault diagnosis by learning domain-invariant features through an optimal mapping function $f_{s}$, but they typically rely on the availability of multiple source domains. However, in the context of compound faults under source-scarce conditions, the effectiveness of these methods is fundamentally limited. The presence of imperfect labels hinders the learning of sufficient domain-invariant knowledge from limited data, as the poor supervisory signal $y^{s}$ cannot adequately guide the feature learning process across domains.

### 2.2. Limitation of Hard Labels and Label Smoothing

**Figure 1:**
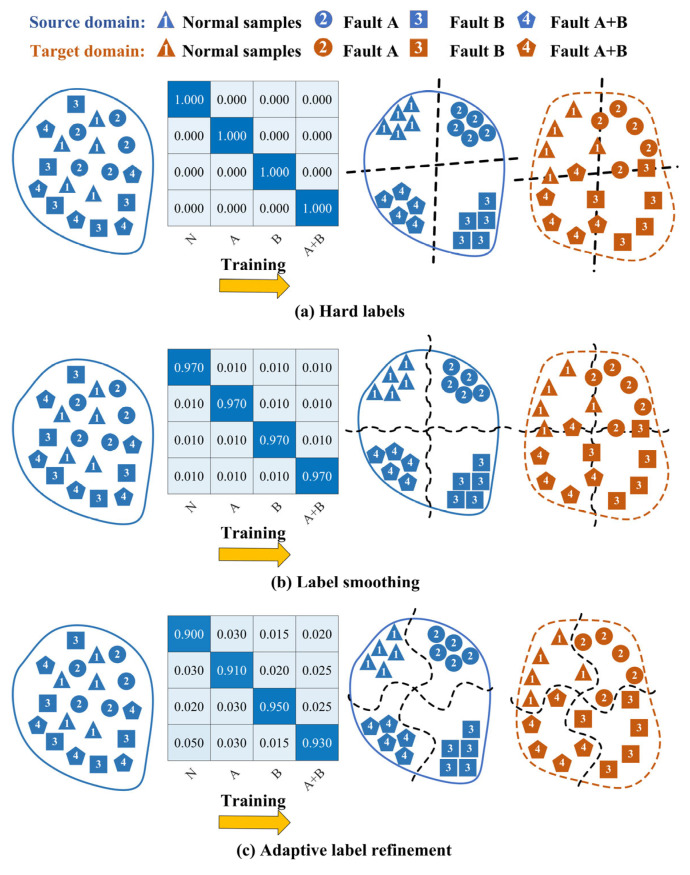
Domain generalization performance of different labeling strategies in compound fault diagnosis.

Hard labels, typically represented as one-hot encoded vectors, compel models to learn discrete and often overly confident decision boundaries [47]. For single faults, high diagnostic accuracy can still be achieved even with a limited number of source domains, owing to their distinct inter-class characteristics and relatively clear decision boundaries. For compound faults, abundant source domains can facilitate high-accuracy diagnosis even with hard labels, as sufficient data enables the learning of transferable domain-invariant features. However, diagnosing compound faults under limited source domains remains a formidable challenge, as it confronts the dual obstacles of extracting sufficient domain-invariant features from scarce source domains and distinguishing classes with inherently ambiguous boundaries, as depicted in Figure 1a. Label smoothing has been adopted to soften the categorical boundaries by introducing a uniform smoothing factor to the hard labels. But this method applies a constant smoothing value uniformly across all classes and samples, failing to capture the specific and often asymmetric semantic relationships between different fault types, as illustrated in Figure 1b. While it may slightly improve calibration, it remains ineffective at modeling the nuanced inter-class correlations essential for accurate compound fault diagnosis under domain shift. Consequently, this paper proposes an adaptive label refinement network (ALRN) to achieve more precise and adaptive label softening through a sample-wise weighting algorithm, as shown in Figure 1c.

## 3. Methodology

### 3.1. Adaptive Label Refinement Network (ALRN)

The adaptive label refinement network utilizes intra-class loss weighting to enhance label representation. By iteratively optimizing the labels multiple times and dynamically fusing fault features with cross-condition common features, it achieves adaptive refinement of the fault classification model.

#### 3.1.1. Feature Extraction

The feature extraction module is trained using a preconstructed dataset through a designed convolutional neural network (CNN), whose detailed architecture is shown in Table 1. The network primarily consists of three convolutional layers and three pooling layers for feature extraction, followed by fully connected layers and a Softmax function for pattern recognition. For the discrepancy between the predicted class probability distribution $q={\left[q_{1},q_{2},\ldots ,q_{n}\right]}^{T}$ generated by the CNN and the label distribution $p={\left[p_{1},p_{2},\ldots ,p_{n}\right]}^{T}$, cross-entropy is employed as the quantitative metric, which is defined as follows:

(3) $$L=-\sum_{i=1}^{n}p_{i}\cdot \log(q_{i})$$

where n denotes the number of classes.

#### 3.1.2. Label Refinement Algorithm

The label refinement employs an intra-class loss weighting algorithm to redefine the label. Samples in the training set from the same class are individually fed into the classification model to obtain the probability distribution outputs for all samples within each class. After the k-th label refinement iteration, for the b-th sample among all a samples of the j-th class in the training set, its probabilistic output $q^{\left(k,j,b\right)}$ after forward propagation through the network is obtained:

(4) $$q^{\left(k,j,b\right)}={\left[q_{1}^{\left(k,j,b\right)},q_{2}^{\left(k,j,b\right)},\ldots ,q_{n}^{\left(k,j,b\right)}\right]}^{T}$$

The optimized label $P^{\left(k\right)}$ is defined as:

(5) $$P^{\left(k\right)}=[p^{\left(k,1\right)},p^{\left(k,2\right)},\ldots ,p^{\left(k,n\right)}]={\left[\begin{array}{cccc} p_{1}^{\left(k,1\right)} & p_{1}^{\left(k,2\right)} & \ldots & p_{1}^{\left(k,n\right)}\\ p_{2}^{\left(k,1\right)} & p_{2}^{\left(k,2\right)} & \ldots & p_{2}^{\left(k,n\right)}\\ \vdots & \vdots & \ddots & \vdots \\ p_{n}^{\left(k,1\right)} & p_{n}^{\left(k,2\right)} & \ldots & p_{n}^{\left(k,n\right)} \end{array}\right]}_{n\times n}$$

when k=0, the $P^{\left(0\right)}$ denotes an n×n identity matrix, which corresponds to the conventional hard-label assignment.

For training samples propagated through the neural network, the loss demonstrates a strong positive correlation with the degree of deviation from ideal label assignments. Therefore, larger loss values are assigned greater weights in the probabilistic output. The cross-entropy loss is preferred over the Kullback–Leibler divergence in our weighting scheme due to its balanced weighting behavior. While KL divergence could potentially accelerate convergence by focusing more aggressively on high-loss samples, it approaches zero when predictions match labels and thus risks excluding well-classified samples from contributing to label refinement. In contrast, cross-entropy maintains a finite positive value that preserves the participation of all samples. This design effectively prevents a few potentially erroneous samples from dominating the label refinement process, thereby significantly improving the robustness of the aggregation. Consequently, from Equation (3), the weight for the probabilistic output is expressed as:

(6) $$\alpha^{\left(k,j,b\right)}=-\sum_{i=1}^{n}p_{i}^{\left(k,j\right)}\cdot \log(q_{i}^{\left(k,j,b\right)})$$

Then the label $P^{\left(k+1\right)}$ after the (k+1)-th refinement is represented as:

(7) $$\begin{array}{l} p^{\left(k+1,j\right)}=\frac{\sum_{b=1}^{a}\alpha^{\left(k,j,b\right)}\cdot q^{\left(k,j,b\right)}}{\sum_{b=1}^{a}\alpha^{\left(k,j,b\right)}}\\ P^{\left(k+1\right)}=[p^{\left(k+1,1\right)},p^{\left(k+1,2\right)},\ldots ,p^{\left(k+1,n\right)}] \end{array}$$

where $p^{\left(k+1,j\right)}$ denotes the label for the j-th class after the (k+1)-th label refinement.

The proposed label refinement strategy mitigates the impact of imperfect supervision by adaptively weighting the intra-class probabilistic outputs from a trained CNN to generate calibrated soft labels. Finally, the network undergoes iterative training using the updated labels $P^{\left(k+1\right)}$.

#### 3.1.3. Label Refinement Stability Coefficient

For the b-th sample among all a samples of the j-th class in the training set, the average KL-divergence loss after forward propagation through the CNN model is defined as follows:

(8) $${\bar{K}}_{j}=\frac{\sum_{b=1}^{a}\left(\sum_{i=1}^{n}p_{i}^{\left(j,b\right)}\log\left(\frac{p_{i}^{\left(j,b\right)}}{q_{i}^{\left(j,b\right)}}\right)\right)}{a}$$

Under the assumption of ideal model convergence with near-perfect label assignments, the average KL-divergence values across different classes should be approximately equal or at least of comparable magnitude. Hence, a label refinement stability coefficient β is addressed to quantify the deviation from ideal label assignments, defined as:

(9) $$\beta =\frac{\underset{1\leq j\leq n}{\max}{\bar{K}}_{j}}{\underset{1\leq j\leq n}{\min}{\bar{K}}_{j}}$$

The iteration stops when $\left|\beta_{k}-\beta_{k+1}\right|$ falls below the threshold δ, namely:

(10) $$\left|\beta_{k}-\beta_{k-1}\right|<\delta$$

**Figure 2:**
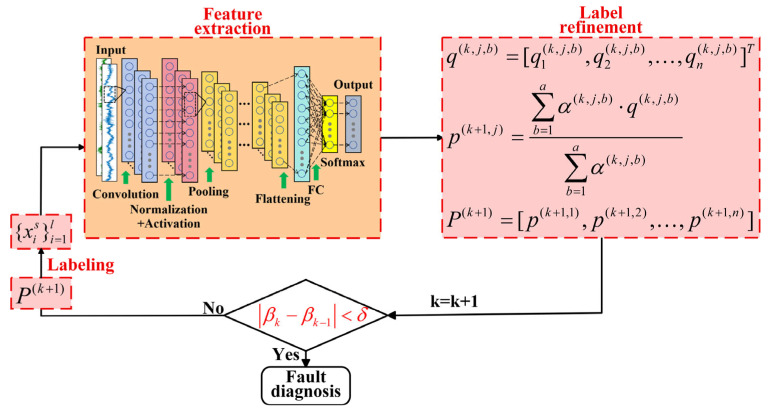
Structure of the ALRN.

The intra-class loss weighting algorithm was shown in Figure 2. And the flowchart of pseudocode describing training process of ALRN was shown in Algorithm 1.
**Algorithm 1** Training and test procedures for ALRN.**① Training:**Input: Labeled source domain dataset ${\left\{x_{i}^{s}\right\}}_{i=1}^{l}$.Set the hyperparameters, including the learning rate, convolutional layers, pooling layers, activation functions, epochs, batch size. Set the labels $P^{\left(0\right)}$ as the n×n identity matrix and threshold δ.1:    **For** k-th label refinement iteration **do**2:           **Let**
$P=P^{k}$.3:           **For** each epoch **do**4:                   Calculate the output q of the model.5:                   Solve loss L based on Equation (3).6:                   Calculate the gradients and update the model parameters.7:           **End for**8:           Calculate weights for the probability distribution outputs based on Equation (6).9:           Slove $P^{\left(k+1\right)}$ based on Equation (7).10:         **If** k=0 **do**11:                 Calculate $\beta_{0}$ based on Equation (9).12:         **Else do**13:                 Calculate $\beta_{k}$ based on Equation (9).14:                 **If** $\left|\beta_{k}-\beta_{k-1}\right|<\delta$ **do**15:                        **Break out**16:                 **End if**17:         **End if**18:    **End for**19:    Save trained model.Output: The trained ALRN diagnostic model.**② Testing:**Feed the target domain samples into model for fault diagnosis. 

### 3.2. Overall Structure

**Figure 3:**
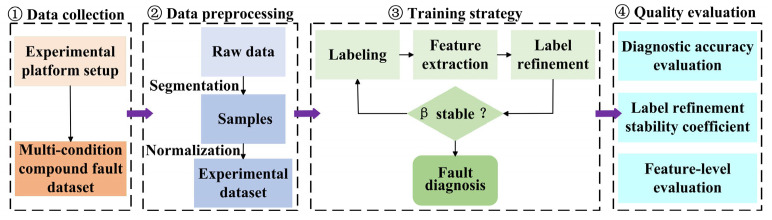
Overall structure of the proposed framework.

Based on the proposed model, the research framework is shown in Figure 3. The procedure begins with establishing an experimental platform for planetary gearbox fault diagnosis to collect acoustic signals under multiple operating conditions and for different fault types. The collected data are then segmented into fixed-length samples and normalized to construct the experimental dataset. Subsequently, a CNN-based feature extraction module is built, and the adaptive label refinement algorithm is executed. This algorithm, guided by the stability coefficient, autonomously determines when the refinement process is sufficient. Finally, the method’s effectiveness is evaluated using both diagnostic accuracy and the proposed stability coefficient.

## 4. Experiments and Results

### 4.1. Data Collection and Description

**Figure 4:**
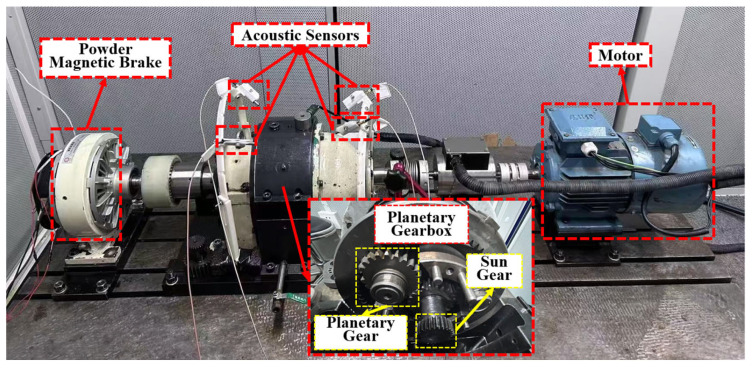
Acoustic signal acquisition platform.

The fault acoustic signals were collected from the planetary gearbox within a semi-anechoic chamber. The experimental setup, illustrated in Figure 4, consisted of a planetary gearbox and an acoustic testing system. By adjusting the variable-frequency motor and powder magnetic brake, the reducer could operate under different conditions including various rotational speeds and torque loads. The acoustic testing system was equipped with four detachable acoustic sensors (Type 46AE, GRAS Sound & Vibration, Holte, Denmark) with a sampling frequency of 10 kHz and a data acquisition device (Model NI 9234, National Instruments, Austin, TX, USA) [48,49].

**Figure 5:**
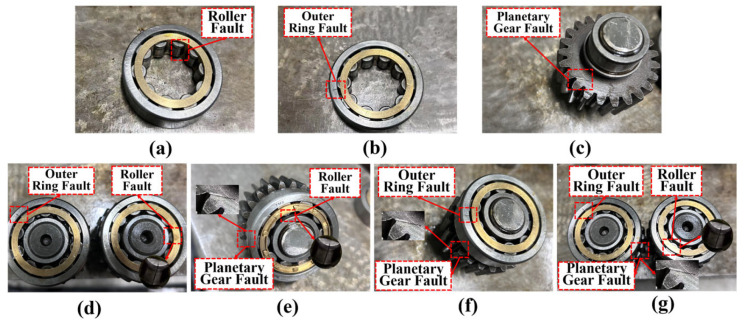
The fault types of planetary gearboxes: (**a**–**c**) Single-component faults; (**d**–**g**) Compound faults.

The planetary gearbox was seeded with seven distinct crack-related fault types, as depicted in Figure 5. These comprised three single-component faults: bearing outer ring (O), bearing roller (R), and planetary gear (P). Four compound faults were also introduced: combined failures of the outer ring and roller (OR), outer ring and planetary gear (OP), roller and planetary gear (RP), and a tri-component failure involving all three elements (ORP). A normal condition (N) without any faults was also included. Data samples under six distinct operating conditions were collected, with each sample containing 4096 data points, as shown in Table 2.

Stochastic Gradient Descent with Momentum (SGDM) was used with an initial learning rate of 0.001. During each refinement phase, single-source domain training was conducted with a batch size of 32 for 200 epochs. The learning rate was reduced from 0.001 to 0.0001 after the first 150 epochs. Similarly, dual-source training was performed with a batch size of 64 for 300 epochs, with the learning rate reduced from 0.001 to 0.0001 after the first 250 epochs. It is noted that the model was continuously trained on the refined labels without parameter reinitialization, thus preserving knowledge from previous stages. Moreover, all algorithms were implemented on a computing platform with an Intel(R) Core(TM) i7-8700 CPU @ 3.20 GHz, 16.0 GB RAM, and an NVIDIA GeForce GTX 1070 GPU.

### 4.2. Performance of Adaptive Label Refinement Network

The domain generalization capability of the proposed Adaptive Label Refinement Network (ALRN) was evaluated through cross-domain fault diagnosis tasks under limited source domain conditions. The experimental framework incorporated two representative scenarios: (1) single-source domain generalization and (2) dual-source domain generalization. Particular attention was devoted to examining the model robustness against significant distribution shifts between source and target domains, especially under substantial rotational speed variations. Table 3 provides the detailed experimental configuration for both scenarios.

**Figure 6:**
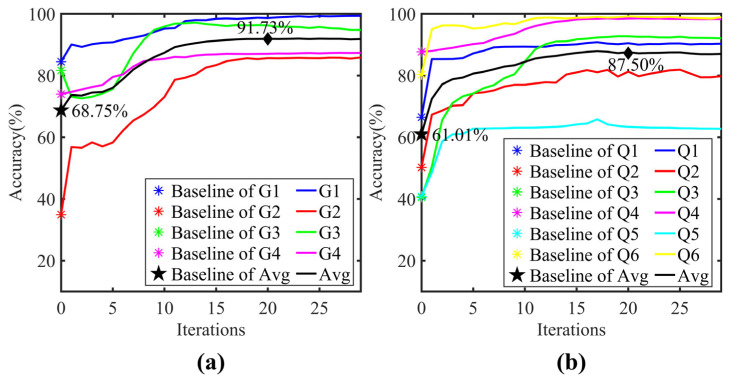
Diagnostic accuracy over refinement iterations: (**a**) Single-source domain; (**b**) Dual-source domain.

**Figure 7:**
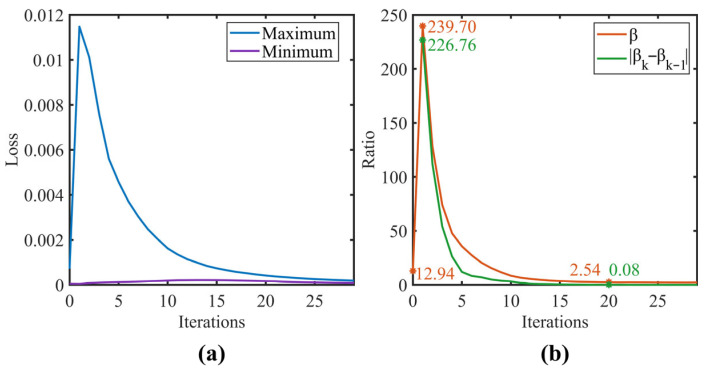
Label refinement stability coefficient for task G1 and G2: (**a**) Bounds of KL-Divergence; (**b**) Maximun/Minimum.

The variation of diagnostic accuracy with refinement iterations is shown in Figure 6. The model diagnostic performance reached its optimum at around 18 to 22 iterations in both scenarios. Compared to the baseline CNN model, the average accuracy after 20 refinement iterations improved significantly from 68.75% to 91.73% for the single-source domain and from 61.01% to 87.50% for the dual-source domain. A slight initial accuracy decrease was observed in task G4 (single-source scenario), but it gradually increased and eventually stabilized as the refinement iterations progressed. Figure 7a illustrates the variation of the maximum and minimum KL-divergence values, while Figure 7b shows the resulting label refinement stability coefficient β for tasks G1 and G2. As depicted in Figure 7b, β underwent a sharp transition from step 0 to 1. This sharp transition was attributed to the initial instability during the hard-to-soft label transition. However, this perturbation did not compromise the overall convergence. After the first iteration, β monotonically decreased from 239.70 and eventually converged to 2.54 at the 20th iteration, coinciding with the peak diagnostic accuracy. The convergence threshold δ was set to 0.1. This value was empirically validated by the convergence behavior observed in our experiments, where the absolute difference in β decreased to 0.08 at the 20th iteration, falling below the predefined threshold. These results demonstrate that the proposed coefficient β effectively quantifies the fidelity of label assignments and its convergence behavior can serve as a reliable criterion for terminating the refinement process.

**Figure 8:**
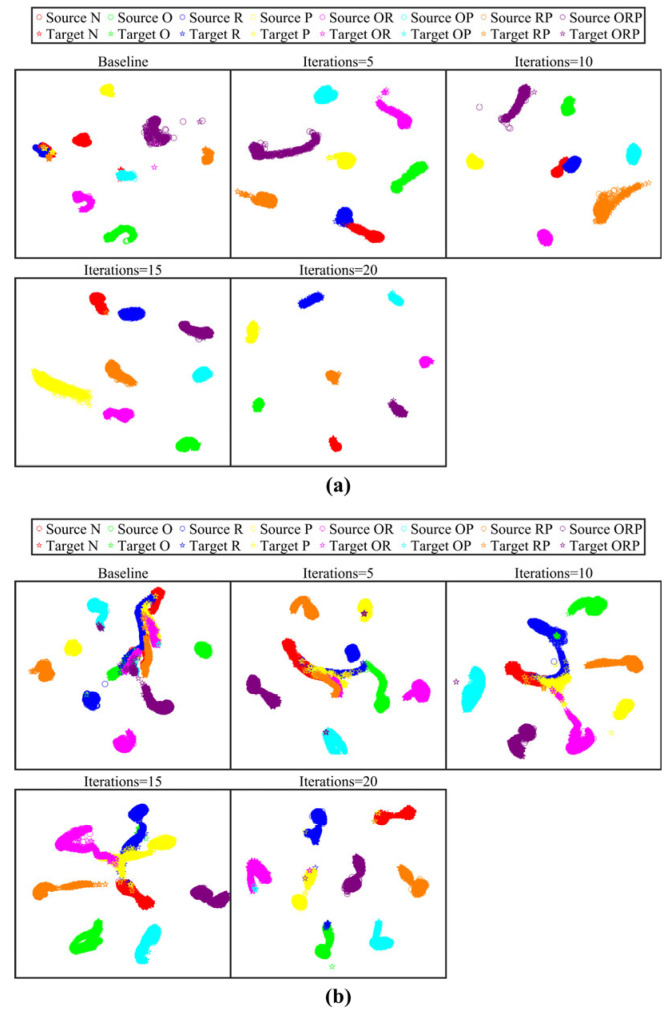
Feature visualization results across label refinement iterations: (**a**) Task G1; (**b**) Task Q3.

To provide an intuitive evaluation, t-SNE [50] was employed to visualize the evolution of feature distributions for single-source (G1) and dual-source (Q3) tasks. Figure 8 shows a comparative analysis between the baseline model (0th iteration) and optimized iterations (5th, 10th, 15th, and 20th), revealing a progressive improvement in class separability. In task G1, the initial iteration exhibited significant cluster overlap between classes N and R. In task Q3, the initial distributions of classes N, O, R, P, and OR were highly entangled. Through successive refinement iterations, the inter-class boundaries became increasingly distinct, achieving nearly linear separability across all fault categories by the 20th iteration. These visual results empirically confirm that the ALRN method effectively mitigates the adverse effects of imperfect label supervision in cross-domain scenarios.

### 4.3. Ablation Studies

**Figure 9:**
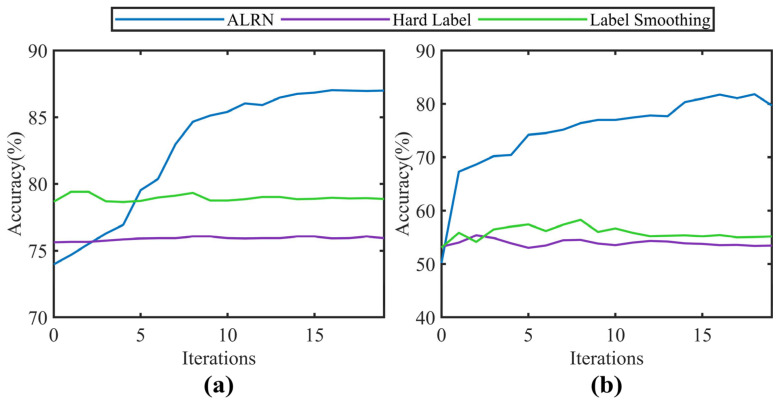
Accuracy of different label weighting strategies across iterations: (**a**) Task G4; (**b**) Task Q2.

An ablation study was conducted to further evaluate the role of the label weighting strategy in the Adaptive Label Refinement Network (ALRN). Figure 9 displays the accuracy trends for ALRN, hard labels, and label smoothing (with a smoothing factor of 0.1) under the same number of iterations on tasks G4 and Q2. The results indicate that while hard labels and label smoothing show no significant change in performance with increasing iterations, only ALRN demonstrates a marked rise in diagnostic accuracy. This outcome substantiates not only the effectiveness of the adaptive label weighting strategy but also the rationality of the selected training cycle, as it ensures that the model is sufficiently trained to converge after each label refinement.

### 4.4. Comparative Experiments

To systematically evaluate the enhancement effect of adaptive label refinement on model cross-domain generalization capability under compound fault conditions and validate the performance superiority of the proposed ALRN, this study conducted comparative experiments with several representative domain generalization approaches, including the classic DANN [28], CORAL [51], MMD [29], and Mixup [52], as well as the recent SDCGAN [41] method, which augments the source domain by generating divergent domains via CycleGAN to learn domain-invariant features through adversarial training, and the MGA-SDG [53] method, which leverages a multi-Gaussian attention mechanism to project multi-scale features into Gaussian spaces for obtaining more consistent and robust feature representations.

**Figure 10:**
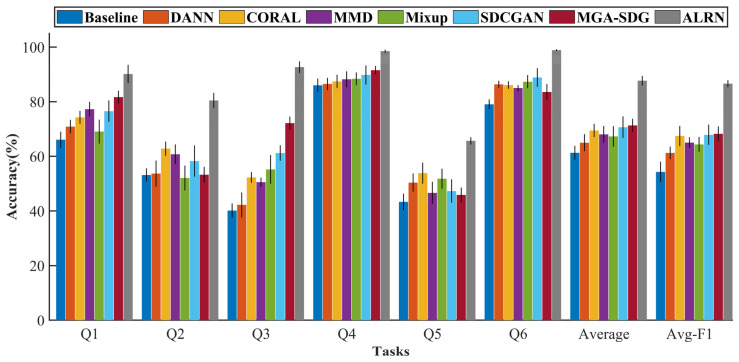
Diagnostic performance comparison different domain generalization methods.

As shown in Table 4 and Figure 10, the proposed ALRN framework demonstrated statistically significant superiority over all benchmark methods across multiple cross-domain tasks. These results were validated through ten independent experimental trials. Each trial was initiated with a different random seed for parameter initialization to ensure the robustness and reliability of our findings. Notably, ALRN achieved near-optimal classification performance in tasks Q4 and Q6, with accuracies of 98.47 ± 0.55% and 98.85 ± 0.31%, respectively. In the challenging scenario of task Q3, it attained a substantially higher accuracy of 92.64 ± 2.11% compared to alternative approaches. A comparative analysis reveals that the classification accuracies in tasks Q2 and Q5 were universally lower for all models investigated. This performance degradation is primarily attributable to the substantial discrepancy in rotational speed between the source and target domains within these specific tasks; namely, a transfer from 900 rpm to 2700 rpm in task Q2, and the reverse from 2700 rpm to 900 rpm in task Q5. Such a significant domain shift exacerbates the difficulty of learning domain-invariant features, thereby presenting a considerable challenge for any domain adaptation method. Crucially, despite these adverse conditions, the proposed ALRN model consistently surpassed all baseline methods, which serves to underscore its superior robustness and enhanced generalization capability in the presence of large domain gaps.

**Figure 11:**
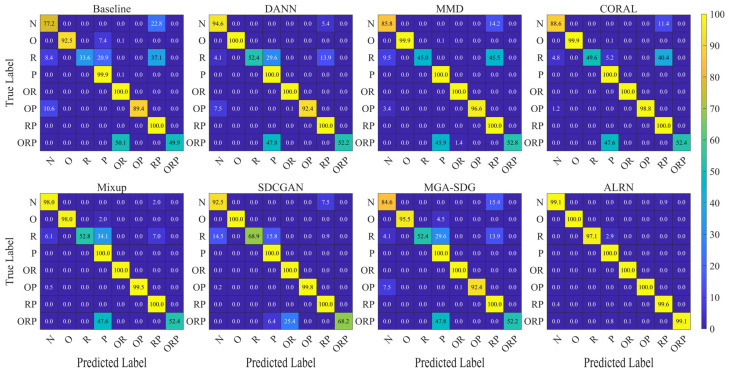
Confusion matrices of different domain generalization methods for task Q6.

**Figure 12:**
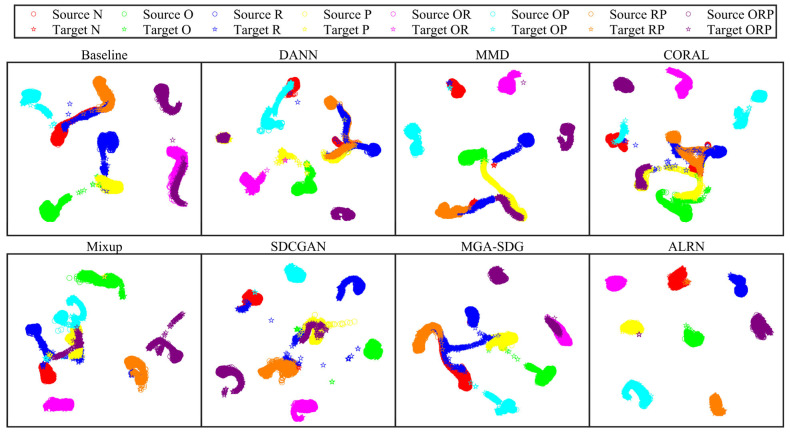
Feature visualization results of different domain generalization methods for task Q6.

The confusion matrix analysis for task Q6, presented in Figure 11, provides further insight into model performance across fault categories. While existing methods, particularly Mixup, showed notable improvement in detecting normal conditions (class N, increasing from 77.2% to 98.0%), their performance remained limited for fault categories R and ORP. In contrast, the ALRN method demonstrated consistent superiority across all fault categories, achieving near-perfect accuracy approaching 100% for classes N, R, and ORP. Feature space visualizations for task Q6, presented in Figure 12, provide complementary structural insight into the model performance. Comparative approaches exhibit substantial feature space overlap among the challenging categories N, R, and ORP. In contrast, the proposed ALRN method establishes clearly separable decision boundaries for all these categories. These visual manifestations corroborate the quantitative results, demonstrating ALRN’s ability to learn discriminative representations that remain invariant under domain shifts. Together, the consistent advantage of ALRN across both quantitative metrics and qualitative visualizations underscores the effectiveness of the adaptive label refinement mechanism in enhancing model generalization under complex compound fault scenarios.

## 5. Discussion

The efficacy of the proposed Adaptive Label Refinement Network (ALRN) has been systematically validated through iterative refinement analysis and comprehensive comparative experiments. The concurrent improvement in diagnostic accuracy and the label refinement stability coefficient across training iterations demonstrates that the adaptive label refinement mechanism effectively mitigates the adverse effects of imperfect supervisory signals, thereby enhancing the model’s generalization robustness under domain shift. Notably, the stability coefficient serves as a novel quantitative metric for assessing label boundary clarity. It achieves this by quantifying inter-class confusion through the disparity in KL-divergence losses across categories under the current label distribution.

Feature space visualizations offer complementary structural evidence for the model’s classification performance. These visualizations reveal that ALRN successfully reduces feature entanglement among easily confusable categories by progressively calibrating the soft label assignments of ambiguous samples. This process leads to more discriminative feature representations and clearer decision boundaries, which are critical for accurate fault diagnosis under distribution discrepancies.

Ablation studies provide further validation of the adaptive weighting mechanism. The observed performance plateau under static label strategies confirms their limitation in refining feature representations, while the progressive accuracy gain achieved by ALRN underscores the critical role of iterative label refinement. This evidence substantiates that the proposed method effectively overcomes the limitations of imperfect labels, enabling the learning of more robust domain-invariant features.

The comparative experiments reveal a notable limitation of existing domain generalization methods such as DANN [28], CORAL [51], and MGA-SDG [53], as their performance is substantially constrained in compound fault diagnosis tasks with scarce source domains. This performance constraint underscores that the imperfection of supervisory signals, which these methods do not explicitly address, becomes a primary bottleneck for generalization in such challenging scenarios. In contrast, ALRN directly addresses the imperfect label issue through its label refinement process, thereby achieving significantly enhanced accuracy and robustness.

Despite its demonstrated advantages, the proposed method is not without limitations. The primary drawback lies in its computational overhead during the training phase. Since ALRN requires k iterative refinement cycles, and each cycle involves a full training pass of the backbone network, the total training time is approximately kT, where T is the time required to train a standard CNN baseline once. This represents a linear increase in costs compared to non-iterative methods. However, this limitation must be evaluated within the context of practical application. Firstly, the training phase is typically offline, where increased computation is an acceptable trade-off for superior performance. Secondly, the inference complexity remains identical to standard CNN. As the iterative refinement is confined to training and does not alter the final model’s architecture, it introduces no additional latency during deployment. This preserves the diagnostic system’s real-time capability, which is crucial for industrial applications.

Furthermore, the current work has three additional aspects that warrant further investigation. Firstly, the convergence threshold δ, while effective in our experiments, was determined empirically. Its generalizability and optimal setting across a wider range of fault types and operational conditions should be explored in future studies. Secondly, the proposed weighting strategy, which is central to the label refinement process, is designed for and validated on high-quality datasets. Its performance may degrade in the presence of significant noise. Therefore, a critical future direction is to investigate the integration of robust data pre-processing techniques, such as data denoising or sample screening, with the ALRN framework to ensure its reliability in more diverse and challenging industrial environments. Finally, the experimental validation in this study is primarily based on data from planetary gearboxes. While the results are promising, the generalizability of ALRN to other critical rotating machinery (such as bearings and motors) under compound fault conditions remains to be further verified. Expanding its scope of application is another key objective for our future research.

## 6. Conclusions

This article proposes a novel Adaptive Label Refinement Network (ALRN) for compound fault diagnosis. The method tackles the limited domain generalization capability arising from the inherent limitations of hard labels and label smoothing. Its core is a progressive label refinement algorithm that mitigates the impact of imperfect supervision, thereby enhancing the model’s ability to learn transferable and discriminative features in cross-domain scenarios. A key innovation is the KL-divergence-based refinement stability coefficient, which objectively measures label assignment quality and provides a principled criterion for terminating the refinement process.

Experimental results demonstrate ALRN’s superior diagnostic performance under data-scarce conditions, outperforming conventional domain generalization approaches that typically require abundant source domains. This advance is particularly significant for industrial applications where source domains are severely limited. Although ALRN incurs greater computational overhead during training, it maintains the high inference efficiency of conventional methods, fully meeting the real-time requirements of industrial fault diagnosis systems.

Future work will focus on three key directions to further enhance the practical applicability of the method. Firstly, we aim to optimize training efficiency, facilitating potential online learning applications. Secondly, the generalizability of the ALRN will be systematically evaluated on compound fault datasets from other critical components of rotating machinery, such as bearings and motors. Lastly, we will rigorously assess the robustness of the algorithm in real-world industrial settings, characterized by significant acoustic noise and interference, which is essential for transitioning ALRN from a laboratory solution to a reliable industrial diagnostic tool.

## Figures and Tables

**Table 1:** Structure of feature extraction module.

Layer Type	Output Size	Kernel Size	Padding Type/Stride	Activation Type	Pool Type/BN
Input	1 × 4096 × 1				
Conv1D_1	1 × 4096 × 32	1 × 16	same	ReLU	BN
Pool1D_1	1 × 1024 × 32	1 × 4	4		Maxpool
Conv1D_2	1 × 1024 × 64	1 × 8	same	ReLU	BN
Pool1D_2	1 × 256 × 64	1 × 4	4		Maxpool
Conv1D_3	1 × 256 × 128	1 × 4	same	ReLU	BN
Pool1D_3	1 × 1 × 128	1 × 256			Avgpool
Output	8 × 1			Softmax	

**Table 2:** Settings of different operating conditions.

Domain	Rotational Speed	Load Torque	Number of Samples Per Class	Total Number of Samples
A0	900 r/min	1 Nm	400	3200
A1	900 r/min	1 Nm	400	3200
B0	1800 r/min	2 Nm	400	3200
B1	1800 r/min	2 Nm	400	3200
C0	2700 r/min	3 Nm	400	3200
C1	2700 r/min	3 Nm	400	3200

**Table 3:** Fault diagnosis experimental configurations.

Scenario	Tasks	Source Domain	Sample Size	Target Domain	Sample Size
Single-source domain	G1	B0	3200	A0	3200
	G2	B0	3200	C0	3200
	G3	B1	3200	A1	3200
	G4	B1	3200	C1	3200
Dual-source domain	Q1	A0, A1	6400	B0, B1	6400
	Q2	A0, A1	6400	C0, C1	6400
	Q3	B0, B1	6400	A0, A1	6400
	Q4	B0, B1	6400	C0.C1	6400
	Q5	C0, C1	6400	A0, A1	6400
	Q6	C0, C1	6400	B0, B1	6400

**Table 4:** Diagnosis result of different domain generalization methods.

Model	Q1	Q2	Q3	Q4	Q5	Q6	Average	Avg-F1
Baseline	66.07 ± 2.91	53.17 ± 2.41	40.14 ± 2.58	86.02 ± 2.42	43.34 ± 2.96	79.03 ± 1.75	61.30 ± 2.51	54.27 ± 3.67
DANN	70.90 ± 2.46	53.66 ± 4.76	42.23 ± 4.53	86.46 ± 2.25	50.39 ± 3.28	86.36 ± 1.27	65.00 ± 3.10	61.26 ± 2.22
CORAL	74.28 ± 2.37	62.80 ± 2.57	52.28 ± 1.91	87.41 ± 2.41	53.84 ± 3.79	86.10 ± 1.33	69.45 ± 2.40	67.45 ± 3.68
MMD	77.29 ± 2.64	60.75 ± 3.59	50.52 ± 1.68	88.21 ± 2.88	46.63 ± 3.98	84.96 ± 1.09	68.06 ± 3.03	65.05 ± 1.88
Mixup	69.05 ± 4.36	52.04 ± 4.50	55.21 ± 5.22	88.40 ± 2.30	51.80 ± 3.65	87.29 ± 2.38	67.30 ± 3.74	64.37 ± 2.67
SDCGAN	76.54 ± 3.87	58.27 ± 5.65	61.21 ± 2.78	89.78 ± 3.45	47.29 ± 4.26	88.89 ± 3.38	70.66 ± 3.90	67.88 ± 3.67
MGA-SDG	81.67 ± 2.28	53.29 ± 2.87	72.14 ± 2.32	91.54 ± 1.49	45.87 ± 2.66	83.52 ± 2.89	71.34 ± 2.42	68.27 ± 2.69
**ALRN**	**90.11 ± 3.32**	**80.43 ± 2.79**	**92.64 ± 2.11**	**98.47 ± 0.55**	**65.65 ± 1.27**	**98.85 ± 0.31**	**87.69 ± 1.73**	**86.57 ± 1.27**

## Data Availability

Data will be made available on request.
